# Transactions of American Dental Association

**Published:** 1882-08

**Authors:** 


### Transactions of American Dental Association.

TWENTY-FIRST ANNUAL SESSION, held at New York, July, 1881.

This is a neat volume of 212 pages, containing the papers, volunteer and others, read before the different sections of the Association. The discussions, upon some of the papers, are given in full. Also, minutes of business transactions, and list of all the officers since the first meeting.

This volume is of real interest and value for those who attended the meeting. It will preserve in convenient form for reference all items of special interest at the time, and to those who were absent it will perhaps serve to arouse a desire to participate in meetings which must be always a means of advancement for the profession. It is to be hoped that all dentists will desire to have this work in their own libraries. Orders may be sent to Dr. George H. Cushing, Chicago, Ill.

The Southern Dental Association will meet in the Baltimore College of Dental Surgery, cor. of Eutaw and Franklin Sts., on the 8th of August, next.

